# Supplementary material for: Relationship Between Facial Melasma and Ocular Photoaging Diseases
Source: Med Sci (Basel). 2025 May 16;13(2):61. doi: 10.3390/medsci13020061 (PMC12101396; doi:10.3390/medsci13020061)
Supplement: Supplementary file 1 [file medsci-13-00061-s001.zip › medsci-3535731-supplementary.pdf]

Supplementary Tables

Supplementary Table S1. Typical clinical presentations of facial melasma and ocular photoaging diseases.

Diseases or conditions	Clinical presentations	References
Facial melasma	Common features include brown to bluish-gray discoloration with either sharp or blurred margins, along with symmetrical patches of irregular brown to gray-brown pigmentation on areas such as the cheeks, forehead, nasal bridge, upper lip, jawline, and upper arms. The predominant distribution patterns are malar, involving the cheeks and nose, and centrofacial, covering the forehead, cheeks, nose, upper lip, and chin.	[1, 2, 68, 69]
Pinguecula	A yellowish, mildly elevated thickening of the conjunctiva located near the corneal margin, most often on the nasal side.	[70]
Pterygium	A triangular-shaped fibrovascular overgrowth of the conjunctiva that extends onto the corneal surface.	[71]
Climatic droplet keratopathy	Progressive anterior corneal haziness begins with limbal microdroplets, followed by central band-shaped subepithelial opacity, and culminates in yellow subepithelial deposits, potentially impairing vision.	[30]
Cataract	Opacification of the crystalline lens reduces light transmission to the retina and leads to visual impairment. The main types include nuclear, which begins at the center and is commonly age-related; cortical, which presents as spoke-like opacities in the lens cortex; and posterior subcapsular, which forms at the back of the lens and particularly affects near vision.	[72, 73]
Age-related macular degeneration	Characterized by small, yellow, round deposits known as drusen at the level of the retinal pigment epithelium, along with areas of the retinal pigment epithelium hyperpigmentation and atrophy.	[74]

Supplementary Table S2. Univariate logistic regression analysis with respect to the presence of pinguecula.

Characteristics	B	Crude OR	95% CI	p value
Age (years)	0.04	1.041	1.023 - 1.059	<0.001
Gender (female=1, male=2)	0.104	1.11	0.619 - 1.988	0.727
BMI (kg/m^2)	0.016	1.016	0.952 - 1.084	0.631
Fitzpatrick skin type				
Type III	NA	1.000	NA	NA
Type IV	0.109	1.115	0.632 - 1.968	0.707
Type V	0.274	1.315	0.419 - 4.125	0.639
Microtrauma (yes=1, no=0)	0.222	1.248	0.737 - 2.115	0.410
Hypertension (yes=1, no=0)	0.606	1.832	1.024 - 3.278	0.041
Diabetes mellitus (yes=1, no=0)	0.018	1.019	0.503 - 2.064	0.959
Dyslipidemia (yes=1, no=0)	0.324	1.383	0.809 - 2.363	0.236
Thyroid disorders (yes=1, no=0)	-0.491	0.612	0.179 - 2.093	0.434
Dry eye (yes=1, no=0)	0.437	1.549	0.655 - 3.661	0.319
Related medications				
Statins (yes=1, no=0)	0.147	1.158	0.679 - 1.976	0.589
Anticonvulsants (yes=1, no=0)	NA	NA	NA	NA
Smoking (yes=1, no=0)	NA	NA	NA	NA
Alcohol consumption (yes=1, no=0)	0.725	2.064	0.695 - 6.132	0.192
Family history of melasma (yes=1, no=0)	0.082	1.086	0.633 - 1.861	0.765
Wearing sunglasses when outdoors (yes=1, no=0)	0.431	1.539	0.913 - 2.594	0.105
Presence of melasma (yes=1, no=0)	0.344	1.411	0.826 - 2.411	0.207
SEPI Part I	0.012	1.012	0.951 - 1.077	0.698
SEPI Part II	0.006	1.006	0.943 - 1.072	0.866

Note. B, regression coefficient; CI, confidence interval; NA, not applicable due to low sample size or as the reference category; OR, odds ratio; SEPI, The Sun Exposure and Protection Index.

Supplementary Table S3. Multivariate logistic regression analysis with respect to the presence of pinguecula.

Characteristics	B	Adjusted OR	95% CI	p value
Age (years)	0.048	1.050	1.030–1.080	<0.001
Gender (female=1, male=2)	-0.378	0.685	0.317–1.510	0.340
BMI (kg/m^2)	0.019	1.020	0.950–1.090	0.603
Fitzpatrick skin type				
Type III	NA	1.000	NA	NA
Type IV	-0.264	1.020	0.950–1.090	0.603
Type V	-0.189	0.768	0.393–1.510	0.440
Microtrauma (yes=1, no=0)	0.134	1.140	0.633–2.080	0.658
Hypertension (yes=1, no=0)	0.387	1.470	0.721–3.080	0.294
Diabetes mellitus (yes=1, no=0)	-0.331	0.718	0.325–1.660	0.423
Dyslipidemia (yes=1, no=0)	0.594	1.810	0.585–6.230	0.139
Thyroid disorders (yes=1, no=0)	-0.731	0.481	0.127–2.070	0.294
Dry eye (yes=1, no=0)	0.530	1.700	0.707–4.600	0.261
Related medications				
Statins (yes=1, no=0)	-0.952	0.386	0.106–1.250	0.126
Anticonvulsants (yes=1, no=0)	NA	NA	NA	NA
Smoking (yes=1, no=0)	NA	NA	NA	NA
Alcohol consumption (yes=1, no=0)	0.965	2.620	0.749–11.000	0.152
Family history of melasma (yes=1, no=0)	0.426	1.530	0.827–2.900	0.181
Wearing sunglasses when outdoors (yes=1, no=0)	0.174	1.190	0.644–2.180	0.576
Presence of melasma (yes=1, no=0)	-0.033	0.967	0.506–1.810	0.918
SEPI Part I	-0.007	0.993	0.865–1.140	0.920
SEPI Part II	0.015	0.985	0.852–1.140	0.838

Note. B, regression coefficient; CI, confidence interval; NA, not applicable due to low sample size or as the reference category; OR, odds ratio; SEPI, The Sun Exposure and Protection Index.

Supplementary Table S4. Univariate logistic regression analysis with respect to the presence of pterygium.

Characteristics	B	Crude OR	95% CI	p value
Age (years)	0.030	1.031	1.012 - 1.049	0.001
Gender (female=1, male=2)	0.261	1.299	0.786 - 2.145	0.308
BMI (kg/m^2)	-0.012	0.988	0.934 - 1.046	0.688
Fitzpatrick skin type				
Type III	NA	1.000	NA	NA
Type IV	0.53	1.699	1.038 - 2.781	0.035
Type V	0.09	1.094	0.416 - 2.875	0.856
Microtrauma (yes=1, no=0)	0.406	1.501	0.947 - 2.378	0.084
Hypertension (yes=1, no=0)	-0.134	0.875	0.541 - 1.416	0.587
Diabetes mellitus (yes=1, no=0)	-0.764	0.466	0.233 - 0.931	0.031
Dyslipidemia (yes=1, no=0)	0.221	1.247	0.786 - 1.980	0.348
Thyroid disorders (yes=1, no=0)	0.197	1.218	0.378 - 3.929	0.741
Dry eye (yes=1, no=0)	-0.648	0.523	0.246 - 1.114	0.093
Related medications				
Statins (yes=1, no=0)	0.068	1.070	0.672 - 1.705	0.775
Anticonvulsants (yes=1, no=0)	NA	NA	NA	NA
Smoking (yes=1, no=0)	0.179	1.196	0.443 - 3.233	0.724
Alcohol consumption (yes=1, no=0)	-0.888	0.411	0.162 - 1.042	0.061
Family history of melasma (yes=1, no=0)	-0.403	0.668	0.413 - 1.082	0.101
Wearing sunglasses when outdoors (yes=1, no=0)	-0.040	0.961	0.604 - 1.528	0.866
Presence of melasma (yes=1, no=0)	0.482	1.620	0.985 - 2.664	0.058
SEPI Part I	0.028	1.028	0.974 - 1.086	0.317
SEPI Part II	0.009	1.009	0.954 - 1.067	0.759

Note. B, regression coefficient; CI, confidence interval; NA, not applicable due to low sample size or as the reference category; OR, odds ratio; SEPI, The Sun Exposure and Protection Index.

Supplementary Table S5. Multivariate logistic regression analysis with respect to the presence of pterygium.

Characteristics	B	Adjusted OR	95% CI	p value
Age (years)	0.036	1.040	1.010–1.060	0.002
Gender (female=1, male=2)	0.658	1.930	0.955-3.950	0.068
BMI (kg/m^2)	-0.012	0.988	0.925–1.050	0.713
Fitzpatrick skin type				
Type III	NA	1.000	NA	NA
Type IV	0.509	1.660	0.923–3.010	0.090
Type V	0.032	1.030	0.308–3.320	0.958
Microtrauma (yes=1, no=0)	0.540	1.720	1.020–2.900	0.042
Hypertension (yes=1, no=0)	-0.560	0.571	0.308–1.050	0.072
Diabetes mellitus (yes=1, no=0)	-1.070	0.343	0.153–0.725	0.007
Dyslipidemia (yes=1, no=0)	0.695	2.000	0.689–6.040	0.205
Thyroid disorders (yes=1, no=0)	1.020	2.770	0.720–10.200	0.125
Dry eye (yes=1, no=0)	-0.693	0.500	0.210–1.110	0.099
Related medications				
Statins (yes=1, no=0)	-0.510	0.601	0.195–1.810	0.365
Anticonvulsants (yes=1, no=0)	NA	NA	NA	NA
Smoking (yes=1, no=0)	0.181	1.200	0.334–4.310	0.778
Alcohol consumption (yes=1, no=0)	-1.430	0.239	0.070–0.720	0.015
Family history of melasma (yes=1, no=0)	-0.462	0.630	0.359–1.090	0.102
Wearing sunglasses when outdoors (yes=1, no=0)	0.056	1.060	0.613–1.830	0.842
Presence of melasma (yes=1, no=0)	0.358	1.430	0.810–2.560	0.221
SEPI Part I	0.079	1.080	0.954–1.230	0.218
SEPI Part II	-0.104	0.902	0.789–1.030	0.123

Note. B, regression coefficient; CI, confidence interval; NA, not applicable due to low sample size or as the reference category; OR, odds ratio; SEPI, The Sun Exposure and Protection Index.

Supplementary Table S6. Univariate logistic regression analysis with respect to the presence of cortical cataract.

Characteristics	B	Crude OR	95% CI	p value
Age (years)	0.042	1.043	1.017 - 1.070	0.001
Gender (female=1, male=2)	0.233	1.262	0.685 - 2.326	0.455
BMI (kg/m^2)	0.045	1.046	0.978 - 1.120	0.190
Fitzpatrick skin type				
Type III	NA	1.000	NA	NA
Type IV	0.261	1.298	0.697 - 2.418	0.411
Type V	1.254	3.505	1.324 - 9.280	0.012
Microtrauma (yes=1, no=0)	-0.260	0.771	0.432 - 1.375	0.378
Hypertension (yes=1, no=0)	0.019	1.019	0.564 - 1.842	0.949
Diabetes mellitus (yes=1, no=0)	0.483	1.621	0.801 - 3.283	0.179
Dyslipidemia (yes=1, no=0)	0.446	1.562	0.885 - 2.757	0.124
Thyroid disorders (yes=1, no=0)	-0.147	0.863	0.184 - 4.047	0.852
Dry eye (yes=1, no=0)	-0.302	0.739	0.295 - 1.852	0.519
Related medications				
Statins (yes=1, no=0)	0.376	1.456	0.824 - 2.574	0.196
Anticonvulsants (yes=1, no=0)	NA	NA	NA	NA
Smoking (yes=1, no=0)	-1.352	0.259	0.034 - 1.990	0.194
Alcohol consumption (yes=1, no=0)	0.137	1.147	0.445 - 2.956	0.777
Family history of melasma (yes=1, no=0)	-0.577	0.562	0.300 - 1.051	0.071
Wearing sunglasses when outdoors (yes=1, no=0)	0.117	1.124	0.630 - 2.006	0.692
Presence of melasma (yes=1, no=0)	0.292	1.340	0.721 - 2.488	0.355
SEPI Part I	0.056	1.058	0.989 - 1.132	0.103
SEPI Part II	0.051	1.052	0.983 - 1.127	0.145

Note. B, regression coefficient; CI, confidence interval; NA, not applicable due to low sample size or as the reference category; OR, odds ratio; SEPI, The Sun Exposure and Protection Index.

Supplementary Table S7. Multivariate logistic regression analysis with respect to the presence of cortical cataract.

Characteristics	B	Adjusted OR	95% CI	p value
Age (years)	0.045	1.050	1.010–1.080	0.005
Gender (female=1, male=2)	0.083	1.090	0.459–2.470	0.846
BMI (kg/m^2)	0.047	1.050	0.966–1.140	0.255
Fitzpatrick skin type				
Type III	NA	1.000	NA	NA
Type IV	0.254	1.290	0.624–2.630	0.487
Type V	1.420	4.150	1.220–1.430	0.022
Microtrauma (yes=1, no=0)	-0.430	0.650	0.341–1.220	0.183
Hypertension (yes=1, no=0)	-0.749	0.473	0.223–0.971	0.045
Diabetes mellitus (yes=1, no=0)	0.266	1.300	0.576–2.840	0.511
Dyslipidemia (yes=1, no=0)	0.168	1.180	0.368–3.700	0.773
Thyroid disorders (yes=1, no=0)	0.029	1.030	0.146–4.570	0.972
Dry eye (yes=1, no=0)	-0.194	0.824	0.284–2.080	0.699
Related medications				
Statins (yes=1, no=0)	0.175	1.190	0.370–4.000	0.772
Anticonvulsants (yes=1, no=0)	NA	NA	NA	NA
Smoking (yes=1, no=0)	-2.040	0.130	0.006–0.849	0.075
Alcohol consumption (yes=1, no=0)	0.081	1.080	0.295–3.680	0.899
Family history of melasma (yes=1, no=0)	-0.465	0.628	0.308–1.240	0.188
Wearing sunglasses when outdoors (yes=1, no=0)	0.217	1.240	0.647–2.420	0.519
Presence of melasma (yes=1, no=0)	0.065	1.070	0.541–2.160	0.854
SEPI Part I	0.001	1.000	0.859–1.160	0.988
SEPI Part II	0.006	1.010	0.861–1.180	0.938

Note. B, regression coefficient; CI, confidence interval; NA, not applicable due to low sample size or as the reference category; OR, odds ratio; SEPI, The Sun Exposure and Protection Index.

Supplementary Table S8. Univariate logistic regression analysis with respect to the presence of posterior subcapsular cataract.

Characteristics	B	Crude OR	95% CI	p value
Age (years)	0.075	1.077	1.024–1.133	0.004
Gender (female=1, male=2)	0.658	1.930	0.750–4.971	0.173
BMI (kg/m^2)	-0.014	0.986	0.878–1.108	0.816
Fitzpatrick skin type				
Type III	NA	1.000	NA	NA
Type IV	1.530	4.620	1.559–13.695	0.006
Type V	1.898	6.671	1.466–30.344	0.014
Microtrauma (yes=1, no=0)	0.139	1.149	0.454–2.911	0.769
Hypertension (yes=1, no=0)	0.31	1.363	0.531–3.494	0.519
Diabetes mellitus (yes=1, no=0)	-0.032	0.969	0.272–3.454	0.961
Dyslipidemia (yes=1, no=0)	0.009	1.009	0.394–2.581	0.985
Thyroid disorders (yes=1, no=0)	NA	NA	NA	NA
Dry eye (yes=1, no=0)	-1.005	0.366	0.048–2.820	0.335
Related medications				
Statins (yes=1, no=0)	0.093	1.097	0.429–2.808	0.847
Anticonvulsants (yes=1, no=0)	NA	NA	NA	NA
Smoking (yes=1, no=0)	-0.028	0.972	0.122–7.749	0.979
Alcohol consumption (yes=1, no=0)	0.666	1.947	0.532–7.124	0.314
Family history of melasma (yes=1, no=0)	0.207	1.230	0.480–3.151	0.667
Wearing sunglasses when outdoors (yes=1, no=0)	-0.488	0.614	0.242–1.555	0.303
Presence of melasma (yes=1, no=0)	0.115	1.122	0.414–3.040	0.821
SEPI Part I	0.102	1.108	0.992–1.237	0.070
SEPI Part II	0.108	1.114	0.999–1.243	0.052

Note. B, regression coefficient; CI, confidence interval; NA, not applicable due to low sample size or as the reference category; OR, odds ratio; SEPI, The Sun Exposure and Protection Index.

Supplementary Table S9. Multivariate logistic regression analysis with respect to the presence of posterior subcapsular cataract.

Characteristics	B	Adjusted OR	95% CI	p value
Age (years)	0.045	1.050	1.010–1.080	0.005
Gender (female=1, male=2)	0.083	1.090	0.459–2.470	0.846
BMI (kg/m^2)	0.047	1.050	0.966–1.140	0.255
Fitzpatrick skin type				
Type III	NA	1.000	NA	NA
Type IV	0.254	1.290	0.624–2.630	0.487
Type V	1.420	4.150	1.220–1.430	0.022
Microtrauma (yes=1, no=0)	-0.430	0.650	0.341–1.220	0.183
Hypertension (yes=1, no=0)	-0.749	0.473	0.223–0.971	0.045
Diabetes mellitus (yes=1, no=0)	0.266	1.300	0.576–2.840	0.511
Dyslipidemia (yes=1, no=0)	0.168	1.180	0.368–3.700	0.773
Thyroid disorders (yes=1, no=0)	0.029	1.030	0.146–4.570	0.972
Dry eye (yes=1, no=0)	-0.194	0.824	0.284–2.080	0.699
Related medications				
Statins (yes=1, no=0)	0.175	1.190	0.370–4.000	0.772
Anticonvulsants (yes=1, no=0)	NA	NA	NA	NA
Smoking (yes=1, no=0)	-2.040	0.130	0.006–8.490	0.075
Alcohol consumption (yes=1, no=0)	0.081	1.080	0.295–3.680	0.899
Family history of melasma (yes=1, no=0)	-0.465	0.628	0.308–1.240	0.188
Wearing sunglasses when outdoors (yes=1, no=0)	0.217	1.240	0.647–2.420	0.519
Presence of melasma (yes=1, no=0)	0.065	1.070	0.541–2.160	0.854
SEPI Part I	0.001	1.000	0.859–1.160	0.988
SEPI Part II	0.006	1.010	0.861–1.180	0.938

Note. B, regression coefficient; CI, confidence interval; NA, not applicable due to low sample size or as the reference category; OR, odds ratio; SEPI, The Sun Exposure and Protection Index.

Supplementary Table S10. Univariate logistic regression analysis with respect to the presence of age-related macular degeneration.

Characteristics	B	Crude OR	95% CI	p value
Age (years)	0.082	1.086	1.015 - 1.162	0.017
Gender (female=1, male=2)	0.387	1.472	0.420 - 5.158	0.545
BMI (kg/m^2)	-0.065	0.937	0.798 - 1.101	0.432
Fitzpatrick skin type				
Type III	NA	1.000	NA	NA
Type IV	0.490	1.632	0.486 - 5.484	0.428
Type V	NA	NA	NA	NA
Microtrauma (yes=1, no=0)	0.824	2.280	0.654 - 7.953	0.196
Hypertension (yes=1, no=0)	1.214	3.365	0.963 - 11.758	0.057
Diabetes mellitus (yes=1, no=0)	-0.677	0.508	0.064 - 4.057	0.523
Dyslipidemia (yes=1, no=0)	0.919	2.506	0.718 - 8.743	0.150
Thyroid disorders (yes=1, no=0)	NA	NA	NA	NA
Dry eye (yes=1, no=0)	-0.386	0.679	0.085 - 5.455	0.716
Related medications				
Statins (yes=1, no=0)	1.001	2.72	0.780 - 9.494	0.117
Anticonvulsants (yes=1, no=0)	NA	NA	NA	NA
Smoking (yes=1, no=0)	0.588	1.800	0.217 - 14.942	0.586
Alcohol consumption (yes=1, no=0)	NA	NA	NA	NA
Family history of melasma (yes=1, no=0)	0.343	1.409	0.420 - 4.721	0.579
Wearing sunglasses when outdoors (yes=1, no=0)	-0.946	0.388	0.111 - 1.355	0.138
Presence of melasma (yes=1, no=0)	0.327	1.387	0.360 - 5.338	0.635
SEPI Part I	-0.012	0.988	0.855 - 1.142	0.874
SEPI Part II	-0.034	0.967	0.828 - 1.129	0.669

Note. B, regression coefficient; CI, confidence interval; NA, not applicable due to low sample size or as the reference category; OR, odds ratio; SEPI, The Sun Exposure and Protection Index. Due to the small number of events in the dependent variable (3.5% of the sample), multivariate logistic regression could not be reliably performed. The extreme class imbalance, combined with the limited number of positive cases, led to model instability and separation issues. To avoid biased estimates, only univariate analyses were conducted, acknowledging the potential confounding effects of unadjusted covariates.

Supplementary Table S11. Univariate logistic regression analysis with respect to the presence of facial melasma.

Characteristics	B	Crude OR	95% CI	p value
Age (years)	0.029	1.03	1.013–1.047	<0.001
Gender (female=1, male=2)	0.087	1.09	0.651–1.852	0.745
BMI (kg/m^2)	0.066	1.068	1.006–1.137	0.033
Fitzpatrick skin type				
Type III	NA	1.000	NA	NA
Type IV	1.005	2.731	1.584–4.864	<0.001
Type V	0.766	2.150	0.797–6.827	0.154
Microtrauma (yes=1, no=0)	0.302	1.352	0.844–2.182	0.212
Hypertension (yes=1, no=0)	0.044	1.045	0.643–1.713	0.861
Diabetes mellitus (yes=1, no=0)	0.731	2.078	1.051–4.427	0.044
Dyslipidemia (yes=1, no=0)	0.586	1.798	1.110–2.947	0.018
Thyroid disorders (yes=1, no=0)	0.45	1.568	0.457–7.179	0.507
Dry eye (yes=1, no=0)	0.346	1.414	0.694–3.072	0.357
Related medications				
Statins (yes=1, no=0)	0.408	1.504	0.930–2.461	0.100
Anticonvulsants (yes=1, no=0)	NA	NA	NA	NA
Smoking (yes=1, no=0)	0.917	2.502	0.795–11.029	0.157
Alcohol consumption (yes=1, no=0)	0.329	1.390	0.615–3.443	0.448
Family history of melasma (yes=1, no=0)	0.438	1.550	0.95–2.561	0.083
Wearing sunglasses when outdoors (yes=1, no=0)	0.107	1.113	0.693–1.784	0.656
SEPI Part I	0.019	1.019	0.963–1.078	0.516
SEPI Part II	0.019	1.019	0.962–1.081	0.529

Note. B, regression coefficient; CI, confidence interval; NA, not applicable due to low sample size or as the reference category; OR, odds ratio; SEPI, The Sun Exposure and Protection Index.

Supplementary Table S12. Multivariate logistic regression analysis with respect to the presence of facial melasma.

Characteristics	B	Adjusted OR	95% CI	p value
Age (years)	0.034	1.034	1.013–1.057	0.002
Gender (female=1, male=2)	-0.324	0.723	0.346–1.520	0.389
BMI (kg/m^2)	0.078	1.081	1.012–1.158	0.023
Fitzpatrick skin type				
Type III	NA	1.000	NA	NA
Type IV	1.124	3.078	1.657–5.923	0.001
Type V	0.681	1.976	0.620–7.153	0.268
Microtrauma (yes=1, no=0)	0.239	1.27	0.744–2.179	0.382
Hypertension (yes=1, no=0)	-0.511	0.6	0.316–1.130	0.115
Diabetes mellitus (yes=1, no=0)	0.493	1.637	0.748–3.840	0.234
Dyslipidemia (yes=1, no=0)	1.232	3.429	1.199–10.872	0.027
Thyroid disorders (yes=1, no=0)	0.756	2.129	0.544–10.685	0.306
Dry eye (yes=1, no=0)	0.298	1.348	0.614–3.120	0.468
Related medications				
Statins (yes=1, no=0)	-0.996	0.369	0.111–1.112	0.087
Anticonvulsants (yes=1, no=0)	NA	NA	NA	NA
Smoking (yes=1, no=0)	0.920	2.51	0.649–12.594	0.211
Alcohol consumption (yes=1, no=0)	0.306	1.359	0.454–4.287	0.590
Family history of melasma (yes=1, no=0)	0.691	1.996	1.131–3.600	0.019
Wearing sunglasses when outdoors (yes=1, no=0)	0.019	1.019	0.579–1.786	0.947
SEPI Part I	-0.032	0.968	0.851–1.102	0.622
SEPI Part II	0.023	1.024	0.897–1.167	0.726

Note. B, regression coefficient; CI, confidence interval; NA, not applicable due to low sample size or as the reference category; OR, odds ratio; SEPI, The Sun Exposure and Protection Index.
